# Analysis of Strategies to Increase User Retention of Fitness Mobile Apps during and after the COVID-19 Pandemic

**DOI:** 10.3390/ijerph191710814

**Published:** 2022-08-30

**Authors:** Jae-Yoon Kwon, Ji-Suk Lee, Tae-Seung Park

**Affiliations:** 1 Department of Fitness MBA, Sangmyung University, Seoul 03016, Korea; 2 Department of Dance & Performance, Hanyang University, 55, Ansan-si 15588, Gyeonggi-do, Korea; 3 Department of Physical Education, Sejong University, Seoul 05006, Korea

**Keywords:** consumer behavior, COVID-19, increasing user retention, mobile, fitness app, IPA

## Abstract

The COVID-19 pandemic has changed the fitness-related field. More people started working out at home, and the use of fitness mobile apps that can measure the amount of exercise through a scientific method has increased compared to before the COVID-19 pandemic. This phenomenon is likely to continue even after the COVID-19 pandemic, and therefore this study aimed to investigate the importance of and satisfaction with a fitness app’s functions according to consumers while using the fitness mobile app. Through this study, we intended to provide data for creating an environment where users can use fitness mobile apps consistently. A total of 420 questionnaires were distributed through Google Survey for about 3 months, from 13 September to 20 November 2020, and a total of 399 complete questionnaires were analyzed in this study. Regarding the data processing methods, frequency analysis, exploratory factor analysis, reliability analysis, descriptive statistical analysis, and IPA were used. The results are as follows. First, the first quadrant of the IPA matrix indicated the high importance of and satisfaction with the fitness mobile app, and included five attributes: cost-effectiveness, easy-to-understand information, ease of use and application, privacy protection, and compatibility with other devices. Second, the second quadrant of the matrix indicated relatively low satisfaction in association to high importance and included five attributes: accurate exercise information provision, design efficiency, daily exercise amount setting, convenient icons and interface, and provision of images and videos in appropriate proportions. Third, the third quadrant of the matrix, indicating low importance and low satisfaction, included five attributes: not sharing personal information, overall design composition and color, customer service, reliable security level, and providing information on goal achievement after exercising. Fourth, in the quadrant of the matrix, indicating low importance and high satisfaction, five attributes were included: exercise notification function, continuous service provision, step count and heart rate information, individual exercise recommendation, and individual body type analysis information.

## 1. Introduction

The world is undergoing major changes due to the COVID-19 pandemic that began in early 2020. Mandatory wearing of masks and social distancing policies are in effect in most countries, and many social and economic experts are saying that we need to prepare for new COVID-19 bursts after the pandemic [1,2]. Taking care of one’s own health is becoming more important than ever [3]. In a situation where activities such as exercise in a gym or jogging outdoors are limited, people’s perceptions of health and fitness training have changed greatly, and more people exercise at home; therefore, the use of exercise equipment related to fitness has increased significantly [4]. For example, the sales of fitness-related products mainly used at home increased by 59% compared to 2019, and the number of trademark applications related to training products in the market was 504, an increase of 126% compared to a previous average [5]. This means that personal health care is becoming more important after overcoming COVID-19.

In addition, the use of fitness-related apps increased significantly from March to June 2020, when COVID-19 was spreading rapidly. This confirms the link between the coronavirus and an increased usage of fitness apps. The average number of monthly active users (MAU) of mobile-based fitness apps increased by 40%, and the number of completed exercise programs increased by 50% [6]. Mobile fitness apps that measure exercise volume and intensity are meeting the needs of those who want programs available at commercial facilities. It is expected that public interest in health and well-being will increase, and interest in fitness mobile apps will continue to increase.

The advantages of the fitness mobile app mentioned above are, first, that, you can exercise at home whenever you can regardless of time and place, and second, that you can exercise at a relatively low cost because there are no additional expenses and it is possible to measure the amount of exercise and exercise intensity without face-to-face interaction [7]. Fitness mobile apps with these advantages are highly likely to become new exercise guides for consumers if the number of consumers who want non-face-to-face training or home training is maintained or even increased after the COVID-19 pandemic.

These consumer behavior indicators prove that we have entered an era in which taking care of one’s own health is more important than ever.

For this reason, there is a need for research that can lead to a sustainable fitness mobile apps use by analyzing consumers’ perceptions of mobile fitness apps. Through this study, it is possible to predict consumers’ spending behavior in the health and fitness field in the current COVID-19 pandemic era as well as in the post-COVID-19 era and generate profits from it. In the current situation of the COVID-19 pandemic and in the future after the pandemic ends, there is a possibility that the consumption and demand for services related to fitness mobile apps will gradually increase and accelerate. Therefore, an academic study on the analysis of consumer perceptions and behavioral intentions regarding the use of mobile fitness apps that can lead to a sustainable consumption of fitness mobile apps is very meaningful.

In order to analyze consumer perceptions and behavioral intentions in the field of marketing and business performance analysis, IPA, which evaluates the importance of a product or service that consumers think of and how satisfied they are, is used [8]. As reported [9,10,11], it refers to the importance of products or services according to consumers and measures how satisfied they are [12,13].

Previous studies related to fitness and exercise apps focused on health promotion [14,15], on experiences using fitness mobile apps [16,17], and on exercise effectiveness using fitness mobile and gamification apps [18,19]. However, these studies only considered how effective a fitness mobile app is in promoting the health of people with disabilities or patients and how the various experiences obtained through an application are related to service use. As a result, there is insufficient research on consumers’ perceptions of the use of fitness mobile apps and suggesting continuous usage strategies.

This study evaluated through IPA the aspects that consumers who use fitness mobile apps perceive as important and identified the aspects consumers are satisfied with. In addition, we wished to identify priorities and practical information that need to be improved quickly and easily through the matrix.

Therefore, the purpose of this study was to derive the importance of and satisfaction with internal and external factors of a mobile fitness app based on the user’s experience of it and to provide empirical data that can increase user retention of fitness apps.

## 2. Literature Review

### 2.1. Fitness Mobile Applications

As the public’s interest in health and wellness increases, interest in fitness mobile apps continues to increase. Fitness mobile apps can be defined as tools that create new service values by the use of the ICT technology in health care, fitness, and sports areas [20]. Health- and fitness-related apps are classified in different categories depending on the exercise methods they evaluate and on other functions they may provide such as weight management, diet, and medical information programs based on different smartphone OS environments [21]. A fitness mobile app is a service that provides exercise information to general users or helps them manage their health.

In the era where various apps can be used for exercise due to the spread of smartphones, analyzing the importance of and satisfaction with achieving goals according to the needs and desires of people who use the apps will be essential for improving the service quality of fitness mobile apps [22]. It is necessary to closely analyze the subjective emotional state regarding the extent to which expectations for one’s exercise amount and intensity are satisfied using the fitness mobile app and apply it to daily life. The fitness mobile app used in this study is a method that guides users through the process of performing exercise programs through audio and visual texts.

### 2.2. Continuous Intention to Use

In the acceptance of a new information system or information technology, the intention of continuous use is a very important concept defined as the user’s intention to continue using the app in the future or to recommend it to people around the user [23]. In research on the characteristics of mobile apps, it is defined as a psychological state that occurs before the purchase of an actual product [24]. In the study on the fitness mobile app service, the intention of continuous use is defined as the degree of using or planning to use an app [25]. This study to understand the intention of continuous use of the fitness app is based on effectiveness, efficiency, system, UI and amp, UX, and information security and reliability factors.

### 2.3. User Experience

UX (User Experience) can be defined as any experience that occurs when a user directly or indirectly uses a product, service, system, or technology. UX includes all experiences of touching, operating, and using a specific mobile app [26]. The direct UX is the user’s experience of perception, reaction, behavior, etc., while using the product [27]. The indirect UX is the perception of a situation or product with knowledge gained through the experiences of others or information they provide [28]. Satisfaction with this experience leads to the repurchase of the product or service and satisfies the needs and expectations of users, which has a positive effect on the intention to continuously use the product [29,30,31]. This study will evaluate the importance and satisfaction of a fitness mobile app experience to obtain useful information on users’ continuous use intention.

### 2.4. Importance–Performance Analysis (IPA)

**Figure 1:**
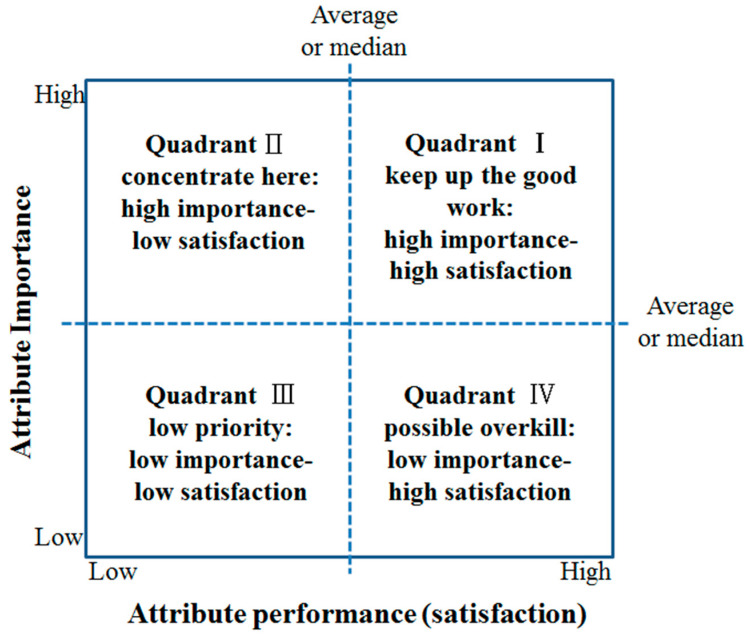
Importance–performance (satisfaction) analysis (IPA) matrix.

IPA analyzes consumer perception through a matrix in four areas: ‘Keep up the good work’ in the first quadrant, ‘Concentrate here’ in the second quadrant, ‘Low priority’ in the third quadrant, and ‘Possible overkill’ in the fourth quadrant [32]. The IPA matrix presented by Boley, MacGehee, and Hammett [32] is shown in Figure 1.

## 3. Method

### 3.1. Participants and Procedure

This study was conducted with the approval of the Research Ethics Committee of Sangmyung University (IRB-SMU-C-2021-9-013). The study targeted adults who had used a particular fitness mobile app, currently available as a free or paid app, within the last six months. The app contained functions that helped the users complete exercises and also provided audio–visual texts. Snowball sampling was utilized to obtain the sample, and a total of 420 questionnaires were distributed between 13 September and 20 November 2020 (i.e., for approximately three months). Considering that face-to-face contact or group activities were difficult during the research planning and process, self-administered questionnaires were distributed online to the selected participants. The participants were able to complete the online survey via a Google Survey URL link sent to their e-mail address or via smartphone. The participants expressed their thoughts and feelings through the questionnaire. Of the collected questionnaires, 21 were rejected because of double entries, inconsistent scores, and omission of answers; thus, 399 questionnaires were finally selected as valid.

Of the total respondents, 70.6% (*n* = 282) were male, and 29.4% (*n* = 117) were female. Regarding the age groups, 46.9% of the participants were in their 20s (*n* = 187), 38.8% in their 30s (*n* = 155), 11.8% in their 40s (*n* = 47), and 2.5% in their 50s and older (*n* = 10). As for period of use, 21.7% had used the app for less than 3 months (*n* = 87), 26.1% for 3–6 months (*n* = 104), 24.9% for 6–9 months (*n* = 99), and 9.5% for more than 1 year (*n* = 38). The purposes of use were as follows: combating lethargy (13.3%, *n* = 53), improving physical strength (26.1%, *n* = 104), controlling weight (33.1%, *n* = 132), strengthening muscles (24.5%, *n* = 98), and others (3.0%, *n* = 12). The frequency of weekly use was as follows: 1–2 times (19.3%, *n* = 77), 2–3 times (46.6%, *n* = 186), 3–4 times (15.8%, *n* = 63), and more than 4 times (18.3%, *n* = 73). Regarding the purchase status, users with free apps accounted for 76.2% (*n* = 304) of the participants, those with paid apps/premium apps accounted for 21.8% (*n* = 87), and those that sometimes paid accounted for 2.0% (*n* = 8).

### 3.2. Measurement

The questionnaire was used as a research tool, and all items and variables were constructed based on previous studies and theories that fit the purpose of the study, and then the survey was conducted by ensuring its content validity. The questionnaire consisted of 46 items divided in three groups: 6 regarded personal characteristics, 20 the perceived importance of fitness mobile apps, and 20 satisfactions with the fitness mobile apps. Since the importance and satisfaction analysis is a useful approach with practical implications, it was judged to be appropriate for this research method. This analysis was modified and supplemented with scales used in research on consumers’ intention to use mobile apps [33,34] and in health care app usability analysis [35]. In addition, items that could not be derived from previous studies or major items that required to be newly derived were added to the survey after a panel of 7 members, consisting of application experts and sports marketing professors, conducted a Delphi survey by going through three stages of structuring the items.

### 3.3. Validity and Reliability of the Survey Tools

Exploratory factor analysis was performed to verify the validity of the questionnaire, which was the research tool of this study. For factor extraction, the eigenvalue was fixed at 1.0 or higher, and only 0.5 or higher was included in the factor loading value. As a result of the exploratory factor analysis, a total of 5 factors were found: Effectiveness, Efficiency, System, UI and UX, and Information Security and Reliability. In addition, the reliability of the survey tool was verified using Cronbach’s α coefficient, and it was judged that the reliability of the survey tool was ensured by a result of 0.774~0.911. Table 1 shows the validity and reliability of this survey tool.

### 3.4. Data Analysis

For the data processing methods of this study, frequency analysis, exploratory factor analysis, reliability analysis, descriptive statistical analysis, and IPA were used with SPSS 25.0 ver.

## 4. Results

### 4.1. Priority Analysis of the Importance and Satisfaction of Fitness Mobile App

Table 2 shows the priority results on the importance attributed by consumers to different aspects related to the fitness mobile apps and on consumers’ satisfaction with them. As for the importance of each attribute, efficiency was judged to be the most important at 3.80, followed by UI and UX at 3.77, system at 3.56, information security and reliability at 3.34, and effectiveness at 3.28. Specifically, in the efficiency factor, ‘content cost performance’, in the UI and UX factor, ‘effective aspect of design configuration’, in the system factor, ‘providing accurate exercise information’, in the security and reliability factor, ‘protecting personal information’, and in the effect factor, ‘personal daily exercise amount setting’ were found to be important. In terms of satisfaction by attribute with the fitness mobile apps, efficiency was found to be the most satisfactory attribute, with a result of 3.60, followed by effectiveness (3.27), information security and reliability (3.22), system (3.19), and UI and UX (2.99). Specifically, the attributes ‘easy to use and quick to learn’ in relation efficiency, ‘providing steps and heart rate’ in relation to in effectiveness, ‘protecting personal information’ regarding information security and reliability, ‘exercise notification function’, in regard to system, and ‘convenient iPhone and interface’ regarding UI and UX showed high satisfaction.

### 4.2. IPA Matrix of Fitness Mobile App

**Figure 2:**
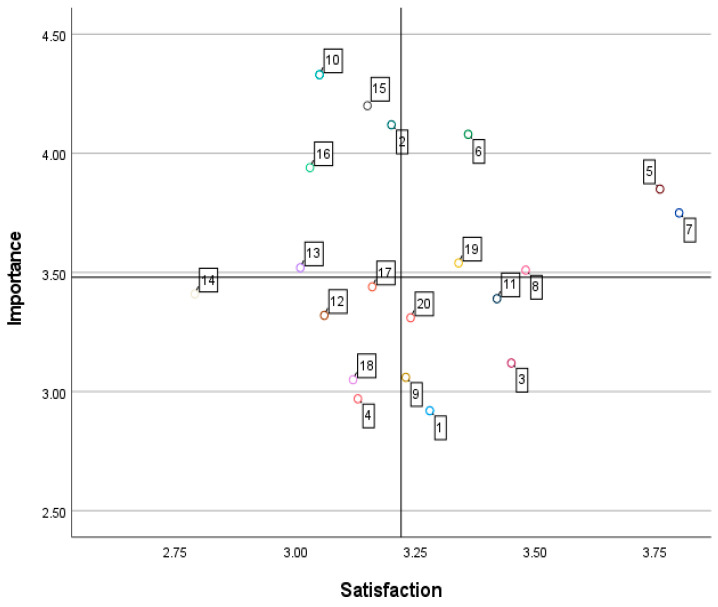
IPA matrix for the fitness mobile app.

IPA analysis was performed to simultaneously analyze the relative importance of and the satisfaction with each attribute of the fitness mobile app. In general, IPA analysis using a matrix is divided into an arbitrary statistical program automatic conversion method and a calculation method by standard deviation for the intersection criterion. In this study, as the most accurate method for setting the center point of each axis, the IP intersection point of importance 3.48 and satisfaction 3.22 was set by centering on the median of the overall mean. In the fourth quadrant, the x-axis indicates the importance, and the y-axis indicates the satisfaction. The analyzed results are shown in Figure 2 and Table 3. First, the first quadrant, which showed high importance of and high satisfaction with the fitness mobile app, contained five attributes: ‘cost performance of content’, ‘easy-to-understand information,’ ‘easy to use and quick to learn’, ‘protecting personal information’, and ‘compatibility with other devices’. Second, the second quadrant, indicating relatively low satisfaction compared to high importance, included five attributes: ‘provide accurate exercise information’, ‘efficient aspect of design configuration’, ‘set individual daily exercise amount’, ‘convenient icon and interface’, and ’proper image and video proportions’. Third, five attributes were included in the third quadrant corresponding to low importance and low satisfaction: ‘not sharing my information’, ‘overall design composition and color’, ‘convenient customer inquiry’, ‘security level reliability’, and ‘providing information about goal achievement after exercise’. Fourth, the fourth quadrant, indicating low importance and high satisfaction, included five attributes: ‘exercise notification function’, ‘continuous service provision’, ‘provide step count and heart rate’, ‘individual exercise recommendation information’, and ‘providing individual body type analysis’.

## 5. Discussion

The purpose of this study was to analyze the importance of using a fitness mobile app and the related perception of satisfaction by using an IPA matrix based on the experiences of users who exercised utilizing the fitness mobile app. As a result of the analysis, the items in the first quadrant of the matrix were attributed high importance and largely satisfied the users and should thus be maintained at the present time.

The first quadrant included a number of attributes related to efficiency, and it can be seen that the current fitness mobile app is convenient to use and satisfactory in relation to the function that makes it easy to understand information it provides. According to a study by Busch, Utesch, Bürkner, and Strauss [14], users reported that the more they perceive that the efficient function of the exercise/fitness app they use has the effect of satisfying their needs, the more positively they rate the app. The results of this study are in partial agreement with our study. Therefore, it is necessary to make efforts to satisfy users’ psychological needs, such as fun, satisfaction, immersion, and interest in using fitness apps [36]. In particular, it is inferred that the efficiency aspect indicating whether consumers can easily understand the information of the fitness mobile app is an important variable that has a positive effect on consumers’ intention to continue using the product; therefore, it should be maintained. In addition, the use of fitness mobile apps is expected to increase in situations where outdoor activities are significantly restricted, as occurred during the COVID-19 pandemic; it is also expected that many people will continue to use these apps even after the end of the pandemic. Therefore, in the case of a fitness mobile app, it is necessary to develop a new business model that can support its continuous use and consumption by consumers by designing a new type of content for mobiles, tablets, PCs, and smart TVs.

The items in the second quadrant of the matrix are of high importance but relatively low satisfaction and can be said to be items that require intensive effort. These items can be interpreted by dividing the research results into two stages. The first stage comprises UI and UX, including design and layout, which are visual elements, such as the overall image of the app obtained through the user experience. It is reported that it is important to expand fitness apps’ accessibility and usability through intuitive design composition and easy UI of exercise, fitness, and other health-related apps that provide voice and visual text [22,37]. UI/UX designs that users think are important but relatively unsatisfying need improvement in usability. In order to do this, a preliminary investigation to improve apps’ usability, centered on the user, will be required. If a heuristic evaluation method is introduced in the initial service model development [38,39], it is possible to find usability problems in the fitness mobile app interface design and to identify consumer-oriented usability in the early stages of UI/UX development. The second research stage is to provide information on personal daily exercise amount and exercise features, set by the user or automatically in the fitness mobile app [40]. In the aforementioned study, the factor that can have a major impact on the psychological satisfaction of exercise participants using fitness apps is the type of coaching provided by the app. This is a prime factor that can replace the need to exercise in fitness centers; it can be said that it is the most important attribute of fitness mobile apps.

In particular, since providing inaccurate exercise-related motions or exercise information leads to injuries, specific instructions should be provided by fitness mobile apps. Every year, more than 60 accidents while exercising using fitness apps are reported [41]. Users should exercise at the appropriate time and intensity of the program presented in the app, but it was found that when exercising alone, users injure their muscles or joints due to inappropriate movements or mistakes. The inability to monitor users’ movements in real time is something that a fitness mobile app must overcome. More effort is required to design apps that can explain exercise movements in more detail to the users.

In addition, it is expected that the satisfaction with fitness app will increase as users can have the same experience as in fitness centers. For example, according to a study by Lee, Kim, Yoon, and Kim [42], users who use a fitness mobile app said that the recorded voice guiding the exercise made them feel as if they were receiving explanations by an actual trainer. Therefore, it is necessary to implement a system that can give users the feeling that a real trainer is watching their exercise, through a convenient interface design and a convincing voice guiding the exercise. It is necessary to improve the system to increase users’ sense of achievement and immersion as well as their interest and fun while exercising by providing information that can prevent injuries, as well as accurate exercise methods.

The items in the third quadrant of the matrix were associated with low importance and satisfaction, indicating that they were not considered a priority. These results stem from users’ high trust in information security due to the nature of mobile apps. In addition, since most fitness mobile apps are services provided by telecommunication companies, users seem to trust them. In relation to this, Millington [43] also found that most users have confidence in the security level when using health and fitness mobile apps, which supports the results of this study to some extent. Therefore, it was found that users do not consider identity theft protection, information about goal achievement after exercise, and customer inquiries much.

However, according to another prior study, as the paid services of fitness mobile apps expand, users are reluctant to use payment methods and mobile payment apps [44]. As fitness mobile apps are widely distributed and popularized, paid services, rather than free apps, are becoming more popular, and many users have concerns about privacy and security issues. Therefore, the current fitness mobile apps or the apps to be released in the future should be based on a personal information security system and improve it as a reliable platform. In addition, it is necessary to reduce the risk of personal information leakage and provide a security system that users can trust.

Quadrant 4 contains items characterized by low importance but high satisfaction; therefore, they need to be considered carefully. Step count, heart rate monitor, and exercise notification functions, which are provided in existing fitness mobile apps, smart watches, and wearable devices, are of low importance to users. In addition, in the case of items provided through various devices, users trust and are highly satisfied with them; therefore, they require continuous attention. However, in the study of Kim and Kim [41], the main users of sports wearable devices or fitness mobile apps are in their 20s and 30s and are interested in new products. That is why these users emphasize the need for an app that provides a variety of information and conforms to the latest fashion or design. Therefore, in addition to providing basic information, items located in the fourth quadrant of the matrix related to the fitness mobile app should provide more specific information on exercise time, recommended daily exercise amount, injury prevention, exercise intensity, and precise exercise movements. In addition, to further satisfy the users, it is necessary to build a platform that provides new information such as behavior tracking, body information monitoring, most appropriate exercise method according to user’s weight, and medical and health information.

## 6. Conclusions and Suggestions

### 6.1. Conclusions

The following conclusions can be drawn from this study. Based on the results of the first quadrant, it was confirmed that users of the fitness mobile app considered many items included in the fitness mobile app as important and were satisfied with them. Therefore, if the app is compatible with various smart devices such as mobile-based devices, tablet PCs, and smart TVs, it is expected that a new business model for the fitness mobile app can be created.

Users value an intuitive use design and easy-to-use user interface, but these attributes were not satisfactory. Therefore, it is necessary to identify the optimal design to improve users’ satisfaction. There is a need to improve the interface design of fitness mobile apps by introducing a heuristic evaluation method for optimal design. In addition, it was confirmed that the provision of accurate exercise movements and exercise information is the most essential element that can make users prefer a fitness app to a fitness center. It is essential to build a system that makes users feel like they are exercising in a fitness center with voice-guided exercises rather than text.

According to the results reported in the third quadrant of the matrix, it was found that users did not consider much security issues, personal goal achievement information, and customer service. However, it was found that, as paid services expand, a reliable personal information protection and security system will be required. Therefore, it is vital to build a personal information security system for fitness mobile apps and provide the related information to users.

Based on the items on the fourth quadrant, it is necessary to maintain the continuous satisfaction of fitness apps users by providing various and specific types of information, in addition to providing basic information. Therefore, specific information such as appropriate exercise time, recommended daily exercise amount, injury prevention, exercise intensity, and accurate motion description should be provided. In addition to that, it is necessary to establish a platform that can offer additional specific information such as activity tracking, body composition, exercise method according to user’s weight, and medical and health information.

### 6.2. Suggestions

Although this study was limited to a fitness mobile app, if a comparative analysis of various other home fitness platforms is made, a business model that combines only the strengths of each platform could be built. Second, this study did not reflect the opinions of the elderly, the disabled, and patients, because it was conducted on young adult men and women who could easily use the mobile app. However, it is expected that more customized fitness app business models will be created if similar research is conducted with other types of users, and the different results are analyzed, compared, and verified. Third, it is necessary to conduct an in-depth study focusing on the motivations of people who use fitness mobile apps. Based on this research, it will be possible to promote healthy lifestyles by developing apps that can be used to increase user retention.

## Figures and Tables

**Table 1:** Questionnaire items and factor loadings from EFA: importance and satisfaction factors of fitness mobile app.

Factor	Items	1	2	3	4	5
Effectiveness	Analysis of personal body type	0.913				
	Setting a personal daily exercise plan	0.906				
	Providing information on step count and heart rate	0.901				
	Providing information on goal achievement after exercise	0.886				
Efficiency	Easy-to-understand information		0.889			
	Cost-effective content		0.872			
	Easy to use and quick to learn		0.842			
	Compatibility with other devices		0.795			
System	Individual exercise recommendations			0.801		
	Provision of accurate exercise information			0.791		
	Exercise notification function			0.757		
	Convenient customer service			0.722		
UI and UX	Appropriate ratio of images and videos				0.794	
	Overall design composition and color				0.773	
	Efficient design configuration				0.757	
	Convenient icon and interface				0.732	
Information Security and Reliability	No sharing of personal information					0.789
	Reliable security level					0.756
	Protection of personal information					0.732
	Status of continuous service provision					0.707
Eigenvalues		3.643	3.302	3.104	2.738	2.685
% of Variance		14.637	13.764	12.594	11.497	9.947
Cumulative %		14.637	28.401	40.995	52.492	62.439
Cronbach’s *α*		0.911	0.898	0.863	0.812	0.774

**Table 2:** Importance, satisfaction, and priority analysis of fitness mobile app.

Factor	Questionnaires	Importance			Satisfaction		
		Rank	*M* ± *SD*		Rank	*M* ± *SD*	
Effectiveness	Analysis of personal body type	20	2.92	0.952	8	3.28	0.894
	Setting a personal daily exercise plan	3	4.12	0.565	11	3.20	0.497
	Providing information on step count and heart rate	16	3.12	0.665	4	3.45	0.584
	Providing information on goal achievement after exercise	19	2.97	0.721	14	3.13	0.395
Efficiency	Easy-to-understand information	6	3.85	0.603	2	3.76	0.486
	Cost-effective content	4	4.08	0.781	6	3.36	0.804
	Easy to use and quick to learn	7	3.75	0.656	1	3.80	0.715
	Compatibility with other devices	10	3.51	0.521	3	3.48	0.961
System	Individual exercise recommendations	17	3.06	0.952	10	3.23	0.676
	Provision of accurate exercise information	1	4.33	0.845	17	3.05	0.707
	Exercise notification function	13	3.39	0.763	5	3.42	0.883
	Convenient customer service	14	3.32	0.561	16	3.06	0.588
UI and UX	Appropriate ratio of images and videos	9	3.52	0.436	19	3.01	0.664
	Overall design composition and color	12	3.41	0.465	20	2.79	0.352
	Efficient design configuration	2	4.20	0.563	13	3.15	0.629
	Convenient icon and interface	5	3.94	0.595	18	3.03	0.618
Information Security and Reliability	No sharing of personal information	11	3.44	0.690	12	3.16	0.547
	Reliable security level	18	3.05	0.786	15	3.12	0.565
	Protection of personal information	8	3.54	0.816	7	3.34	0.653
	Status of continuous service provision	15	3.31	0.772	9	3.24	0.691
Total			3.54			3.25	

**Table 3:** IPA matrix results for the fitness mobile app.

Quadrant	Items
Quadrant 1	Cost-effective content, easy-to-understand information, easy to use and quick to learn, protection of personal information, and compatibility with other devices.
Quadrant 2	Provision of accurate exercise information, efficient design configuration, setting a personal daily exercise plan, convenient icon and interface, and appropriate ratio of images and videos.
Quadrant 3	No sharing of personal information, overall design composition and color, convenient customer service, reliable security level, and providing information on goal achievement after exercise.
Quadrant 4	Exercise notification function, status of continuous service provision, providing information on step count and heart rate, individual exercise recommendation, and analysis of personal body type.

## Data Availability

Not applicable.
